# Normal-tension glaucoma (Low-tension glaucoma)

**DOI:** 10.4103/0301-4738.73695

**Published:** 2011-01

**Authors:** Douglas R Anderson

**Affiliations:** Professor and Distinguished Chair in Ophthalmology, Bascom Palmer Eye Institute, University of Miami Miller School of Medicine, Miami, Florida, USA

**Keywords:** Glaucoma, maximal medical therapy, medical management, target intraocular pressure

## Abstract

Glaucoma is now considered an abnormal physiology in the optic nerve head that interacts with the level of intraocular pressure (IOP), with the degree and rate of damage depending on the IOP and presumably the degree of abnormal physiology. Diagnosis of normal-tension glaucoma (NTG), defined as glaucoma without a clearly abnormal IOP, depends on recognizing symptoms and signs associated with optic nerve vulnerability, in addition to absence of other explanations for disc abnormality and visual field loss. Among the findings are a halo or crescent of absence of retinal pigment epithelium around the disc, bilateral pre-chiasmal visual field defects, splinter hemorrhages at the disc margin, vascular dysregulation (low blood pressure, cold hands and feet, migraine headache with aura, and the like), or a family history of glaucoma. Possibly relevant, is a history of hemodynamic crisis, arterial obstructive disease, or sleep apnea. Neurological evaluation with imaging is needed only for atypical cases or ones that progress unexpectedly. Management follows the same principle of other chronic glaucomas, to lower the IOP by a substantial amount, enough to prevent disabling visual loss. However, many NTG cases are non-progressive. Therefore, it may often be wisein mild cases to determine whether the case is progressive and the rate of progression before deciding on how aggressivene to be with therapy. Efforts at neuroprotection and improvement in blood flow have not yet been shown effective.

## Definition and controversy: The nature of “Normal-Tension” Glaucoma

Glaucoma is now defined as a characteristic type of optic atrophy (with associated loss of visual function), perhaps best characterized by excavation of the optic nerve rather than simple pallor. Its fundamental nature seems to be pathophysiology of the optic nerve, the rate and severity of which is affected by the level of intraocular pressure (IOP). The etiology is poorly understood, but whatever it is, the level of IOP seems to determine whether the etiologic factors will lead to glaucomatous damage or not. About half the people who are found to have an IOP of 35 mm Hg will be found to have developed glaucomatous cupping and field loss.[1] With IOP between 21 and 30 mm Hg, a smaller percentage of individuals will develop glaucoma over several years.[2] Among eyes with IOP in the statistically normal range, the percentage who develop glaucoma is much smaller. However, people whose eyes have a normal IOP are so much more numerous than those with abnormally high pressure that between one-third and one-half of eyes with glaucoma have a “normal” IOP. Thus, an abnormal pressure is not necessarily harmful (hence there are eyes with ocular hypertension, even sometimes with an IOP over 40 mm Hg (unpublished personal observation)), and a normal IOP does not necessarily guarantee against developing glaucoma, hence there are eyes with normal-tension glaucoma (NTG), most often with an IOP between 15 and 20 mm Hg.

As just described, NTG would have no fundamental difference from ordinary chronic primary open angle glaucoma (POAG), except that the etiologic trigger or pathogenic process is accelerated at a lower level of IOP. The implication is that whenever the IOP of an individual is high enough to start the disease, the cascade of pathophysiologic events is the same (such as ischemia, interruption of rapid orthograde and retrograde axonal transport, excessive free radicals, triggering of apoptosis, and collapse of support provided by the lamina cribrosa). In my opinion, the ischemia in glaucoma does not result from simple inadequacy of blood flow, but is due to inadequate *regulation* of blood flow,[3,4] which in the case of the optic nerve may reside in large part in the capillaries,[3,5] and hypothetically with episodes of transient ischemia and re-perfusion injury.[6] The level of IOP that is harmful and the degree of susceptibility is on a continuum, with the number of eyes at risk becoming greater for progressively higher levels of IOP.[1]

A competing viewpoint is that shock-induced optic neuropathy,[7] arteritic anterior ischemic optic neuropathy, or an orbital mass causing atrophy in an eye with a large physiologic cup can produce confusingly similar clinical presentation; so they should each be considered a type of NTG. The analogy is that strokes, high blood pressure, or heart attacks can all have different causes, so the diagnosis of hypertension, stroke, or heart attack does not imply a particular etiologic entity, even if it leads to a final common pathogenic pathway. The diagnosis thus focuses on the final common pathogenic or clinical features, instead of the initiating etiology.

However, in this article the term “pseudo-NTG” will be used to indicate these other causes of cupping and field loss, because it is useful clinically to make a diagnostic distinction among the types in order to guide management. For example, some of these other entities will not be helped in the least by lowering the IOP.[8] Of course, in an analogous manner, it is also true that arterial hypertension is a sign rather than a diagnosis in the true sense, and for the sake of using proper treatment, a distinction is made between hypertension due to eclampsia, hypertension due to renal disease, and essential hypertension. It is similar for glaucoma when there are several types of different etiologies.

## Clinical Manifestations

True NTG has some clinical manifestations that seem associated with particular susceptibility to harm from IOP in the normal range.

1. A region of absent retinal pigment epithelium (RPE) is more often seen as a crescent or halo at the disc border in NTG than in high-pressure glaucoma or normal eyes. The cupping is often worse in the region of absent RPE, with field loss most conspicuous in the corresponding region.[9–11]
2. Features of cupping: In some cases, there is a notch where there is highly localized region of thin or absent neuro-retinal rim, which has sometimes been called a “focal ishcemic” type of cupping.[12] It is associated with a highly localized dense arcuate field defect or even a dense upper hemifield defect. This type is conspicuous on fundus examination, so the presence of NTG is more recognized than when the disc has a less obviously abnormal appearance. Other discs have diffuse shallow cupping and a pale color of the disc and surrounding tissue, leading to the designation as a “senile sclerotic” disc. High myopes may be particularly susceptible to NTG, but their discs are difficult to evaluate. With a frequent temporal crescent, scotomas tend to be closer to fixation than the paracentral scotomas of non-myopes. Still other discs have cupping that resembles ordinary glaucoma with mildly elevated IOP. Most discs, however, cannot be placed into one of these distinctive types, but have a mixture of characteristics. There may be some relationship of the focal ischemic type to vascular dysregulation and of the senile sclerotic type to systemic atherosclerosis.
3. Splinter hemorrhages are reported more frequently in NTG, but may also be seen in uncontrolled POAG.[13] Hemorrhages may simply indicate poor control and thus are more frequent in NTG in which substantial lowering of pressure is more difficult to achieve.
4. POAG and NTG intermingle in the same families, suggesting they are the same or related conditions.
5. Of considerable importance for diagnostic and management decisions is that vascular dysregulation is evident. It may be present in many people with glaucoma, but is strikingly more frequent and more conspicuously symptomatic in those with glaucoma and normal IOP. The manifestations elicited by questioning the patient (or physiologic testing) include cold hands and feet as an over-reaction to cold or stress. Patients may report sleeping with socks on even in warm climates, and noticeably cold hands when greeted with a handshake. Arterial blood pressure tends to be low. Migraine headaches, especially with a visual aura, are more common, and are more frequent in women (which is not true of ordinary POAG). Strangely, although they feel hunger as mealtime approaches, they infrequently or never have a sensation of thirst even when dehydrated.
6. There are reports suggesting that some eyes with signs of glaucoma may be related to an acute ischemic episode (“shock-induced neuropathy”),[7] or chronic obstructive arterial disease,[8,14] which may be non-progressive. This, as well as the relationship of NTG to ischemia from sleep apnea[15] needs further exploration. Cases with particular systemic causes can be considered either a variety of NTG or one type of“pseudo-NTG”.

## Diagnosis

Without an abnormally high IOP to alert the clinician to possible glaucoma, NTG is usually discovered because of the abnormal disc appearance. It is easy to overlook mild glaucomatous cupping during a routine examination unless the patient has a scotoma near or below fixation that prompts a particularly careful examination of the disc and a visual field examination. When the signs of glaucoma are discovered in the absence of an abnormally high IOP, the working diagnosis may be NTG.

Sometimes it is tempting to consider that the diagnosis depends on ruling out all other causes of cupping or field loss. However, it is my preference to look first for evidence that would tend to confirm the diagnosis. This includes items mentioned in the previous section. Is there a prominent crescent or halo of absent RPE? Are splinter hemorrhages observed at the time of recognizing a problem exists or on any visits to monitor the condition before deciding the diagnosis is certain or starting treatment? Is there a first-degree family history of glaucoma? Most productive in my experience is to ask about tendency toward cold hands and feet, low blood pressure, wearing socks at night when sleeping, and migraine headaches. It is reassuring to find signs or symptoms that tend to be associated with NTG. Finally, if the condition is bilateral with field defects that are classical for pre-chiasmal lesions (in particular nerve fiber bundle defects that cross the vertical midline and are not central scotomas), it is difficult to imagine a cause other than glaucoma.

### Differential diagnosis

Contrary to the belief of some, it does not seem necessary to do a neuro-ophthalmic evaluation with neuro-imaging for all cases with an initial working diagnosis of NTG. Yet, it may be wise to consider whether certain other conditions explain the clinical presentation. Re-evaluation becomes wise if the case shows progressive damage despite substantial lowering of the IOP.

Among the clinical characteristics to be considered are:

1. Pseudo-glaucoma:
  - A large physiologic cup with no field loss. I have seen children with a diagnosis of NTG who have simply a large disc with a large physiologic cup. Examination of the parents to discover whether they also have a large physiologic cup is helpful. Some of the new diagnostic instruments will fail to find a thinned retinal nerve fiber layer or a reduced area of the neuro-retinal rim.
  - A congenital anomaly of the disc, perhaps with an arcuate field defect. There may be a notch in the rim of the disc with a colobomatous absence of RPE at the bottom edge of the disc, absent nerve fibers in a wedge from that region, and a corresponding defect. The abnormality can be bilateral. Unilateral cases might have amblyopia since childhood, or even secondary strabismus.
  - Anterior ischemic optic neuropathy. The non-arteritic form typically occurs in eyes with small discs, but if the arteritic form occurs in an eye with a large physiologic cup, the loss of nerve fibers can create a localized thinning or notch in the rim, a feature we have come to associate with glaucoma. The acute episode may be asymptomatic if the affected nerve fibers are not near the fovea. Later the patient may be seen with a large cup and a notched rim, just as in glaucoma, and be associated with a dense field defect in either the upper or lower hemifield. An important clue is that the disc is large. If the disease is unilateral, an erythrocyte sedimentation rate and evaluation for giant cell arteritis might be in order so that treatment might be used to protect the other eye or prevent recurrence in the initially involved eye.
  - Branch retinal vessel occlusion. If the acute event went unnoticed by the patient, there may be an arcuate region of lost nerve fibers and a corresponding visual field defect. If the disc had a physiologic cup of moderate or large size, the neuro-retinal rim may be locally thinned in the region with lost nerve fibers. Of course, an arcuate visual field defect would also be expected.
  - Optic nerve “giant” drusen. Discs with multiple drusen are often obvious. However, we have seen cases in which field defects of nerve fiber bundle topography were found in the presence of a normal IOP. A unilateral case with a tentative diagnosis of NTG was sent for neuro-ophthalmic evaluation, which failed to uncover a cause, so was sent to us for management of NTG. The disc was unusual, however, in having no cupping and did not appear glaucomatous. During careful inspection, a fleeting glisten was seen deep in the disc tissue, and ultimately a small deep drusen was found that corresponded to the location of the visual field defect.[16]
  - Orbital or intracranial tumor. I can recall several cases of a unilateral tumor (such as an orbital meningioma) producing a prechiasmal type of visual field defect. Again, the optic nerves were usually larger than average, with correspondingly large cups. Because of nerve fiber loss, there can be localized thinning of the rim, perhaps subtle, and some pallor that may be difficult to appreciate in the thinned region. Unilateral field defects with symmetry (except for subtle thinning that may be equivocal on examination) of the discs might alert one to this.
2. “High-Pressure” glaucoma
  - Inaccurate tonometry. Applanation tonometers may give a false low reading if the cornea is very thin. Indentation tonometers were known long ago to give false low pressure in large, often myopic, eyes. However, as it has become recognized that NTG is fundamentally the same disease as POAG, and the treatment of both is to lower the IOP, an error of diagnosis on this basis is of little importance.
  - Variable intraocular IOP. The IOP may be abnormally high at certain times of the day, or certain days of the week. Again, the treatment is to lower the IOP, and presumably the treatment will lower the IOP at the times when it is high as well as at the times when it is normal.
  - Past elevation of IOP. Possible causes include chronic use of corticosteroids for contact lens comfort, or uveitis that is no longer active. Chronically intermittent angle closure may also have caused considerable elevation of IOP. Pigmentary glaucoma produces elevated pressure during middle-age years, during which time damage to the optic nerve occurs, but the IOP may return to normal before glaucomatous cupping is found during an eye examination for new glasses or early cataract. Episodes of glaucomatocyclitic crisi may have produced high IOP in the past. Finally, it seems that there may be some cases of “burned out” glaucoma in which changes in aqueous humor dynamics has caused a previously high IOP to return to normal.
    The importance of some of these is that if damage in the past was due to high IOP that is not likely to recur, the condition may now have become stable without the need for treatment.
  - Chronic POAG in which the pressure has been lowered by systemic medication. Beta blockers, for example, while not as effective in lowering IOP when given systemically may, nonetheless, bring the IOP into the normal range.
3. Special forms of NTG
  - Shock-induced neuropathy. Some cases have been identified in which the patient had a severe cardiovascular event in the past,[7] and an excavated optic nerve is recognized on a later routine eye examination. There may also be cases related to chronic atherosclerosis,[8,14] obstructive arterial disease rather than dysregulation. These cases may tend to be non-progressive, if the underlying vascular etiologic cause has been corrected, just as ordinary glaucoma is expected to become stable after the inciting elevation of IOP is lowered. Of possible interest is that magnetic resonance imaging (MRI) evidence of microinfarcts was found in 22% of cases with NTG,[14] the approximate percentage of cases that progressed despite treatment in the collaborative normal-tension glaucoma study,[17] and in keeping with the findings that damage in glaucoma seemed to be related to IOP in the presence of vasospastic disease, but not to blood abnormalities associated with occlusive vascular disease.[8]
  - Hypothetically, there might be a form of NTG that represents a primary optic neuropathy unaffected by IOP. Such cases seem not to have been identified with certainty, but in principle lowering the IOP would be irrelevant in the treatment. These cases may be represented by the rare, frustrating cases that continue to be progressive even after the IOP has been lowered to levels accurately determined to be in the range of 8 to 10 mm Hg at all times.

Initial evaluation: From the description of typical cases of NTG plus the description of entities that might mimic NTG (differential diagnosis), the evaluation is fairly evident. Questions should be asked to discover symptoms of vascular dysregulation (vasospasm), past history of corticosteroid eye drops, history of a hemodynamic crisis, major surgery with a difficult recovery, or a blood transfusion, and family history of glaucoma. Among my favorite questions are whether the patient wears socks to bed at night and whether he was ever so sick that they thought he was going to die, but he lived anyway. Treatment for occlusive arterial disease or sleep apnea, if present, deserves attention whether glaucoma is present or not, and the benefit to glaucoma of attending to these diseases is not known.

Careful ophthalmoscopy may reveal whether the optic disc is characterized by excavation without pallor of the neuro-retinal rim, and likewise whether there are any hints of a congenital anomaly, an old retinal vascular occlusion, an optic nerve head drusen, or other conditions Anterior segment examination may show pigment dusting from previous pigmentary glaucoma or a shallow anterior chamber with narrow angles. Visual field examination should show typical nerve fiber bundle defects.

If the IOP is normal in both eyes, but clearly asymmetric, the optic nerve damage and visual field loss should be correspondingly worse in the eye with higher IOP. However, the glaucomatous damage can be asymmetric even when the IOP is equal, presumably resulting from asymmetry of the abnormal pathophysiology in the optic nerve. However, strictly unilateral cases should be very typical of NTG, or the diagnosis should be questioned.

Neurologic or neuro-ophthalmic consultative evaluation should not be necessary for typical bilateral cases with pre-chiasmal types of visual field defects. However, deviation from a typical clinical presentation or unexpected progression should cause a careful consideration of the diagnosis and perhaps vascular and neurological evaluation. Tests for vascular dysregulation (vasospasm) or arterial disease may help confirm the diagnosis, but treatment is guided by the need to treat these conditions in the first place rather than by any proven benefit for NTG.

Management: If a case seems likely to be of a non-progressive type (history of cardiovascular incident, history of past use of corticosteroid eye drops, evidence of past pigmentary glaucoma, etc.), it may be most reasonable to monitor the patient’s condition until the occurrence and rate of any progression is determined.

If a case seems typical and likely progressive, it seems reasonable to start with one eye and lower the IOP substantially, say from the range of 17-20 mm Hg to 10-13 mm Hg. Decide on the treatment of the second eye when it is evident that the treated eye fared better than the untreated eye with respect to both the disease and any adverse results from treatment.

In uncertain cases, a period of observation may be wise, or non-surgical treatment of one eye, especially if progression is observed and confirmed. The basis for recommending slow initiation of treatment is based on the observations (see below) that many cases do not progress even after years of monitoring. However, if there is severe optic nerve damage and the visual field is greatly affected, it is wise to start treatment. Medical treatment may be started in both eyes, but it may still be wise to evaluate the effectiveness and adverse effects of surgery in one eye of a particular patient before proceeding to the second eye. Even if there is progressive disease, it is likely to be slow with ample time to evaluate the benefit and consequences of surgical treatment aimed at a very low IOP, but not hypotony.

The general principles do not differ from that of managing chronic POAG. Lower the IOP substantially from what is thought to be a damaging level of IOP in that individual, but with enough caution to avoid causing harm in the process. We await evidence that efforts to improve blood flow or enhance its regulation, or to provide neuroprotection (reducing harm despite an insult), is of any benefit in any kind of glaucoma.

### Lessons from recent clinical trials

The Collaborative Normal-Tension Glaucoma (CNTG) study set out to determine whether IOP was or was not involved in the pathogenic process of NTG, which was debated and uncertain in the 1980s. The manner to discover this was to determine whether the course was altered if the IOP was changed, by lowering it by 30%. The main answer was that, yes, lowering the IOP did change the course of the disease.[17] Along the way, various other discoveries were made relevant to NTG and glaucoma in general.[18–25]

To review these secondary outcomes briefly, the desired 30% lowering of IOP can be achieved in patients with NTG with medical therapy and laser trabeculoplasty about half the time, and perhaps this is even more feasible with medications not permitted in the NTG study protocol and with drugs that have more recently become available. In particular, beta blockers were not used in the CNTG study, and prostaglandin analogues were not available.[18] With repeated, frequent visual field examinations in search of very subtle changes or a slow rate of progression, in NTG or likely in any other chronic glaucoma, there is a risk of judging falsely that progression has occurred. Progression must be evident on at least one subsequent field to be sure it is genuine.[19] Once pressure has been successfully lowered 30% from baseline, rate of progressive field loss is slower than in a group that did not receive treatment.[17] Cataracts, which occur more frequently in treated patients who underwent filtration surgery, produce visual changes. In a clinical trial format with visual field as the sole outcome measure, correction for cataract effect must be made to uncover the benefit of lowering the IOP.[20] The rate of visual field progression in cases of untreated NTG is highly variable, some cases showing progression in a few months, but half not showing progression within five years.[21–23] Risk factors involved in the pathogenesis or that can act as prognostic indicators are migraine, female gender, disc hemorrhage at diagnosis, and perhaps racial or genetic heritage.[24] Perhaps not all patients benefit from IOP lowering to the same degree.[25]

The Low-Pressure Glaucoma Treatment Study (LoGTS) is currently underway,[26] with the goal of comparing the stability of NTG patients treated with timolol compared to those treated with brimonidine, but results are not ready to be reported.
